# Retinal alterations resemble brain pathology in a rat model of Parkinson’s disease induced by intranigral infusion of α-synuclein oligomers

**DOI:** 10.1038/s41420-025-02830-0

**Published:** 2025-11-28

**Authors:** Chiara Burgaletto, Anna Flavia Cantone, Maria Francesca Palmas, Chiara Bianca Maria Platania, Giulia Di Benedetto, Gabriella Gaudio, Cristina Barbagallo, Marco Ragusa, Claudio Bucolo, Nunzio Vicario, Ezio Carboni, Alfonso De Simone, Renato Bernardini, Anna R. Carta, Giuseppina Cantarella

**Affiliations:** 1 Department of Biomedical and Biotechnological Sciences, Section of Pharmacology, University of Catania, Catania, Italy; 2 Department of Biomedical Sciences, University of Cagliari, Cagliari, Italy; 3 Department of Biomedical and Biotechnological Sciences, Section of Biology and Genetics, University of Catania, Catania, Italy; 4 Department of Biomedical and Biotechnological Sciences, Section of Physiology, University of Catania, Catania, Italy; 5 Department of Pharmacy, University of Naples “Federico II”, Naples, Italy

**Keywords:** Parkinson's disease, Molecular neuroscience

## Abstract

Parkinson’s disease (PD) is a debilitating neurodegenerative synucleinopathy, characterized by dopaminergic degeneration, pathological deposition of alpha-synuclein (α-Syn), and neuroinflammation in both motor regions of the midbrain and non-motor areas of the cortex. Despite its motor-centric characterization, visual disturbances such as hallucinations, diplopia, altered contrast sensitivity and retinal abnormalities are well-documented non-motor changes of PD. While this evidence points to neuropathological processes in PD that extend beyond the brain, the neuropathological basis of retinal dysfunction and the role of α-Syn remain poorly investigated. Given the central neuropathological role of α-Syn in the PD brain, we assessed whether the retina is affected in a translational rat model of PD based on the intranigral bilateral infusion of toxic oligomers of human α-Synuclein (H-α-SynOs). Rats were stereotaxically injected with H-α-SynOs or PBS (Vehicle) into the substantia nigra pars compacta (SNpc) and sacrificed 3 months post-infusion. Thereafter, several retinal tissue pathological parameters, along with the expression patterns of selected miRNAs and inflammatory markers, were assessed. The retina of rats infused with H-α-SynOs exhibited high levels of phosphorylated-α-Syn (p-α-Syn), along with a significant decrease of tyrosine hydroxylase (TH) expression, reflecting dopaminergic neuron disfunction. Analysis of PD-associated miRNAs in the retina also revealed heightened levels of miR-384-5p, which inversely correlated with the expression of its predicted molecular target, SIRT1, in rats infused with H-α-SynOs. Consistently, H-α-SynOs infusion induced a widespread activation of retinal astrocytes and microglial markers, associated with a heightened proinflammatory cytokine signaling downstream of TLR4/NFκB. Collectively, our data reveal that H-α-SynOs extend their neuropathological effects to retinal damage, reinforcing our rodent model ability to recapitulate PD pathology in both brain and retina. This study underscores the robustness of this preclinical model and its value as translational system for testing proactive interventions targeting PD-related pathology.

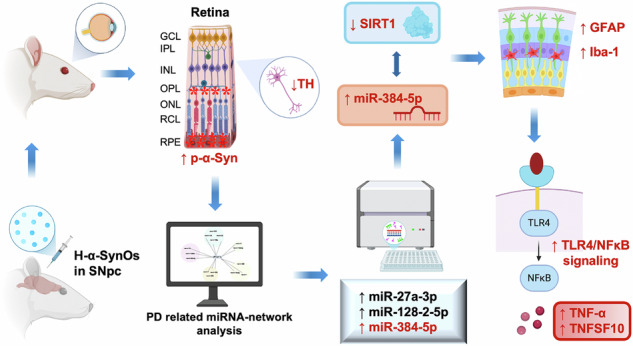

## Introduction

Parkinson’s Disease (PD) is a progressive and chronic neurodegenerative condition, characterized by the degeneration of dopaminergic neurons, the occurrence of alpha-synuclein (α-Syn)-enriched Lewy bodies in the brain, as well as neuroinflammation affecting motor system [1, 2]. Individuals diagnosed with PD experience a range of motor symptoms, including bradykinesia, resting tremor, muscular rigidity and postural instability, mainly attributable to dopaminergic deficiency caused by neuronal loss in the substantia nigra pars compacta (SNpc) [3–5]. Moreover, non-motor signs are common, often preceding motor deficits by many years and becoming increasingly severe as the disease progresses [6]. Notably, visual dysfunction, such as visual hallucinations, diplopia (double vision) [7, 8], low sensitivity to contrast and compromised color perception [9], as well as retinal abnormalities, represent well-documented non-motor symptoms in PD [10]. Intriguingly, as an extension of the central nervous system (CNS), the retina may mirror the brain neuropathology of PD, opening to the investigation of retina for PD diagnosis and disease monitoring.

Retinal structure and function are significantly affected in PD patients, with disease duration significantly impacting on retinal thickness, and with the severity of motor dysfunctions negatively correlating with retinal function [11]. Indeed, PD patients often exhibit retinal layers thinning [12, 13], loss of dopaminergic retinal cells, and the accumulation of α-Syn aggregates in multiple retinal layers [14–16]. While this evidence points to widespread neuropathological processes in PD that extend beyond the brain, the neuropathological basis of retinal dysfunction and the role of α-Syn remain poorly investigated.

Recent studies indicate that soluble aggregates of α-Syn, particularly oligomers and protofibrils, stand as the most toxic species, being detrimental to both neurons and glial cells [17–19]. These toxic aggregates disrupt cellular functions and accelerate neurodegeneration, exacerbating PD progression [20]. In this context, exogenous human α-Syn oligomers (H-α-SynOs) have displayed neurotoxic effects when infused intracerebrally into the SNpc of adult rats, largely featuring the PD phenotype including motor and non-motor symptomatology. Accordingly, in this model, PD-like neuropathology (i.e., dopaminergic neuron degeneration, phosphorylated α-Syn deposits, and neuroinflammation) was observed not only at the infused site but also in distant brain regions, highlighting the spread of α-Syn pathology [21–23].

Inflammation stands as a prominent player in the pathogenesis of PD [24], and a clear association has been established between inflammation and PD risk [19, 25]. Previous studies have established that oligomeric α-Syn elicits an intense immune cells activation in the brain, mainly through binding to Toll-like receptors (TLRs) [21, 22, 26–29]. Similarly, immune cell activation induced by oligomeric α-Syn has been recently described in peripheral blood of PD patients, suggesting that α-Syn pathology and immune dysregulation synergistically contribute to PD pathology both within and beyond the brain [30, 31].

Retinal α-Syn pathology has been investigated in rodents, however previous studies addressed this issue in prodromal models of PD, which do not fully recapitulate the main neuropathological brain features [32]. Therefore, the role of α-Syn toxic species in the retina in PD remains uncertain.

Motivated by human data reporting retinal abnormalities in PD patients and the retina’s integral connection to the CNS, we aimed to determine whether intracerebral infusion of toxic α-Syn could induce retinal pathology characteristic of PD. To address this, we exploited a well-established rat model of PD, induced by the intranigral infusion of H-α-SynOs [21], and investigated whether α-Syn administered into the brain could lead to retinal dysfunction, in addition to the previously documented PD-related pathological changes in the brain.

## Results

### Increased retinal p-α-Syn expression following intracerebral infusion of H-α-SynOs

Considering that α-Syn is a widely distributed protein in PD brain, and that the retina is reported to serve as a mirror reflecting the state of the CNS [10], we investigated the impact of the intranigral infusion of H-α-SynOs on the expression of α-Syn and p-α-Syn in rat retinas.

**Fig. 1:**
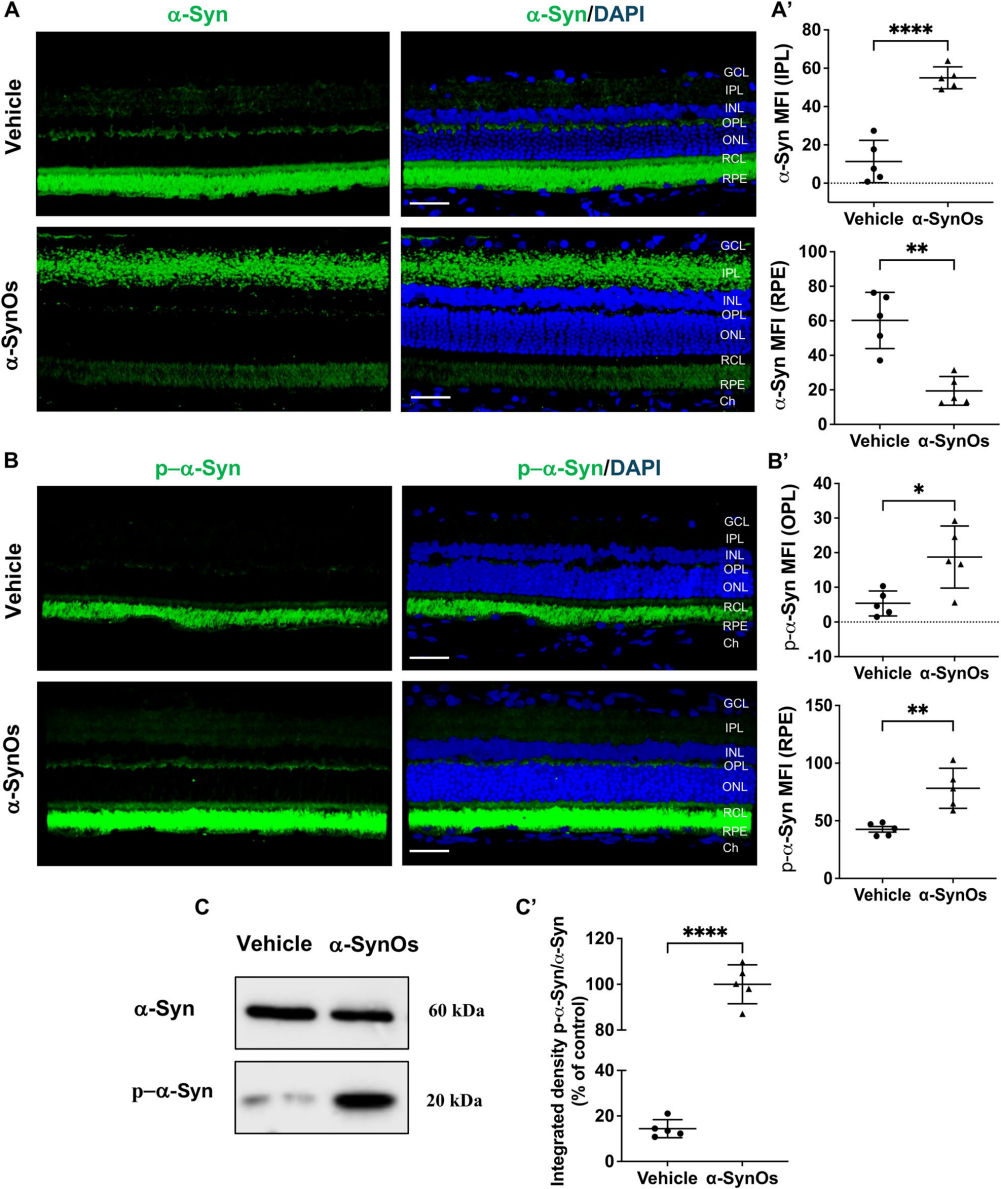
Intracerebral infusion of H-α-SynOs leads to pS129-α-synuclein accumulation in the retina. **A** Confocal microscopy images of retinal slices stained with α-Syn antibody. Original magnification, x40. Scale bar = 50 µm. **A’** Quantification of α-Syn expression in the IPL and RPE from H-α-SynOs or Vehicle infused rats, presented as mean fluorescent intensity (MFI). Data are expressed as means ± SD. *P* values are based on two-tailed unpaired *t* test. **B** Confocal microscopy images of retinal slices stained with pS129-α-synuclein antibody (p-α-Syn). Original magnification, ×40. Scale bar = 50 µm. **B’** Quantification of p-α-Syn expression in the OPL and RPE from H-α-SynOs or Vehicle infused rats, presented as mean fluorescent intensity (MFI). Data are expressed as means ± SD. *P* values are based on two-tailed unpaired *t* test. *N* = 5 animals per group (Vehicle and α-SynOs); five independent retinal samples. **C** Representative western blot images of α-Syn and p-α-Syn protein expression in retinal lysates from H-α-SynOs or Vehicle infused rats. **C’** Densitometric analysis of Western blots. Data are expressed as means ± SD. *P* values are based on two-tailed unpaired *t* test. *N* = 5 animals per group (Vehicle and α-SynOs); five independent retinal samples, two pooled retinas per sample of each group. *p ≤ 0.05, **p ≤ 0.01, ****p ≤ 0.0001.

Immunofluorescence experiments revealed that rats subjected to intracerebral H-α-SynOs infusion showed a significant increase in α-Syn expression within specific retinal layers, particularly the inner plexiform layer (IPL), and a significant decrease in the retinal pigmented epithelium (RPE) layer, compared to the retinas of vehicle-injected rats (Fig. 1A, A’). The principal marker for α-Syn aggregation in PD is phosphorylation at serine 129 (pS129) of this protein [33, 34]. To evaluate whether the intranigral infusion of H-α-SynOs induced pS129-α-synuclein inclusions in the retina, we performed immunohistochemical analyses. Notably, we observed an increased expression of p-α-Syn in the outer plexiform (OPL) and RPE layers of H-α-SynOs infused rats compared to control rats (Fig. 1B, B’).

Consistently with immunohistochemical findings, Western blot (WB) analysis confirmed a significant elevation of p-α-Syn protein expression levels in the retinas of rats receiving intracerebral H-α-SynOs infusion compared to the vehicle-injected rats, relative to basal a-Syn levels (Fig. 1C, C’).

### H-α-SynOs-induced dopaminergic disfunction in the retina

**Fig. 2:**
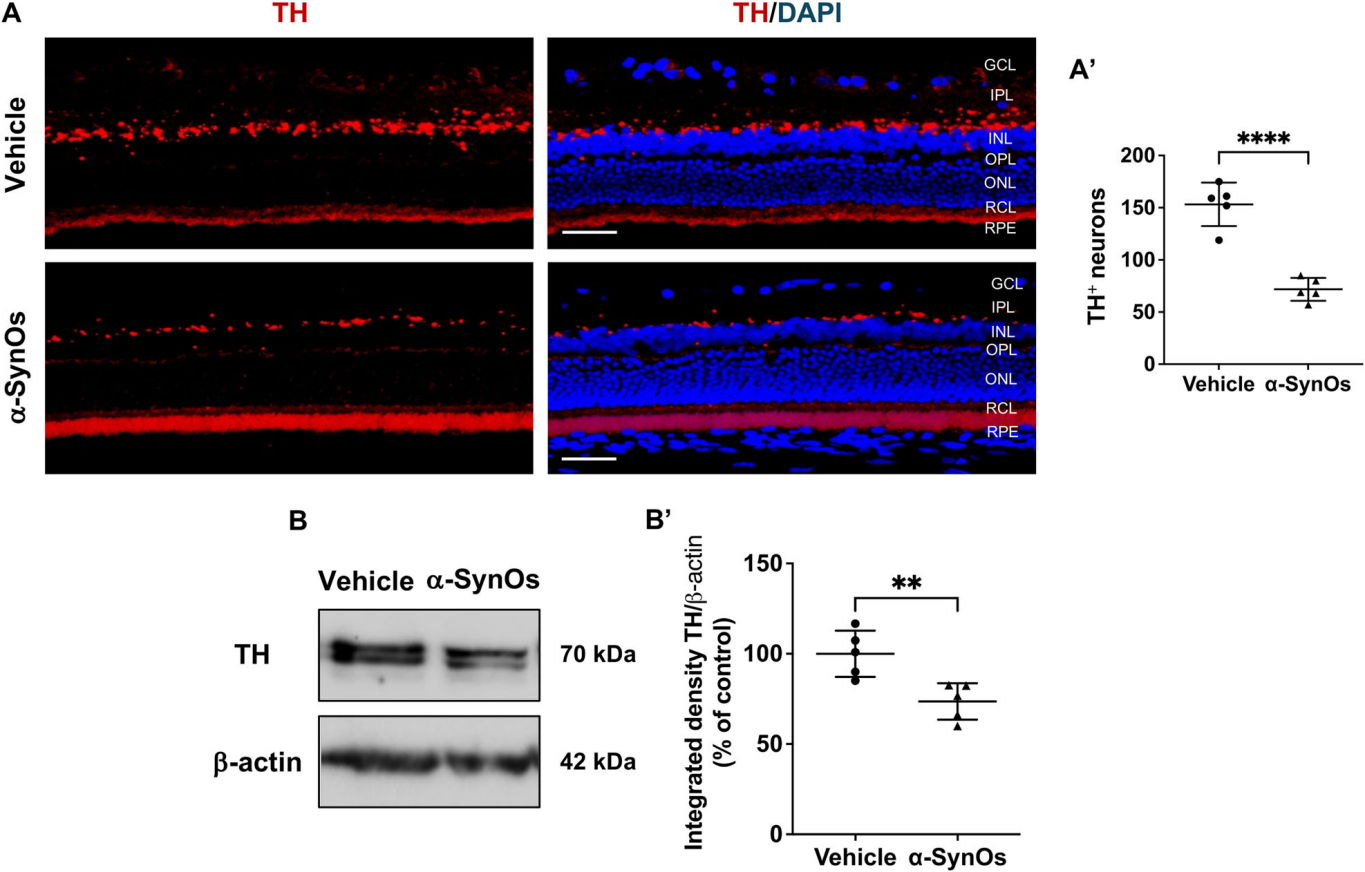
Intracerebral infusion of H-α-SynOs induced dopaminergic neuron disfunction in the retina. **A** Confocal microscopy images of TH-stained retinal slices. Original magnification, x40. Scale bar = 50 µm. **A’** Count of TH+ cells in the retinas from H-α-SynOs or Vehicle-infused rats. Data are expressed as means ± SD. *P* values are based on two-tailed unpaired *t* test. *N* = 5 animals per group (Vehicle and α-SynOs); five independent retinal samples. **B** Representative western blot images of TH protein expression in retinal lysates from H-α-SynOs or Vehicle infused rats. **B’** Densitometric analysis of Western blots. Data are expressed as means ± SD. *P* values are based on two-tailed unpaired *t* test. *N* = 5 animals per group (Vehicle and α-SynOs); five independent retinal samples, two pooled retinas per sample of each group. **p ≤ 0.01, ****p ≤ 0.0001.

Consistently with the heightened levels of retinal p-α-Syn observed in rats receiving intracerebral H-α-SynOs infusion, and the vulnerability of dopaminergic neurons reported in PD [18, 19, 29, 30], we explored the impact of H-α-SynOs infusion on dopaminergic neurons integrity in the retina. To test such changes, we immunostained retinal sections for tyrosine hydroxylase (TH), which catalyzes the rate-limiting step in the biosynthesis of dopamine [35]. We found a significant reduction of neurons expressing TH in the retina of H-α-SynOs-infused rats compared to controls, likely reflecting an impairment in this neuronal population (Fig. 2A, A’). Particularly, this reduction was substantially evident in the IPL of the rat retinas.

Consistently, WB analysis corroborated the results obtained for the TH count, confirming that the H-α-SynOs infusion triggered a significant decrease in TH expression (Fig. 2B, B’).

As a control of H-α-SynOs toxicity against dopaminergic neurons, stereological counting of TH^+^ cells within the SNpc showed a significant reduction in cell number in H-α-SynOs-infused rats as compared to vehicle-treated rats, further supporting the above-mentioned findings (Supplementary Fig. 1).

### Upregulation of predicted PD-associated miRNAs in the retinas of H-α-SynOs-infused rats

**Fig. 3:**
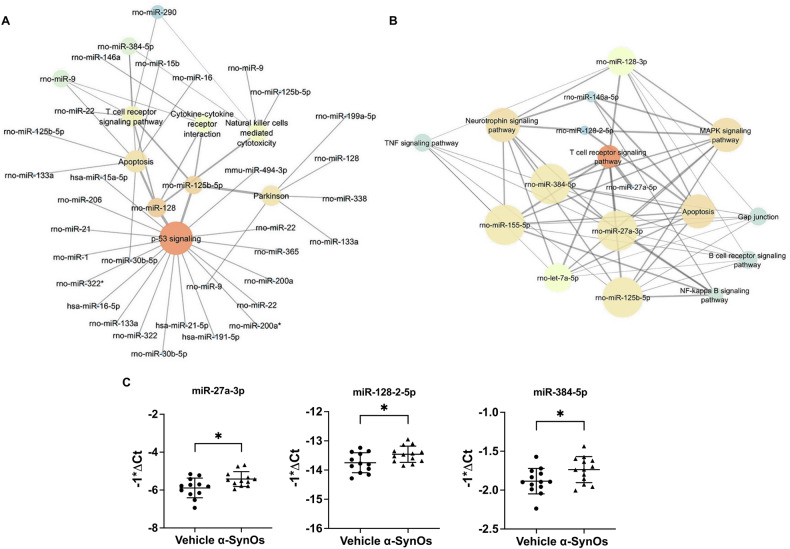
Upregulation of predicted PD-associated miRNAs in the retinas of rats infused with H-α-SynOs. **A** miRNA-to-pathway network predicted by Diana MirPath v.3. **B** pathway-to-miRNA network predicted by Diana MirPath v.3. Node dimensions are directly proportional to node closeness centrality. Node color (from blue to red) is proportional (temperature scale) to node grade value. Edge thicknesses are proportional to edge betweenness values. Centrality metrics analysis of networks was carried out using Cytoscape v.3.7.0. **C** Graphs showing upregulation of predicted PD-associated miRNA in the retinas of rats infused with H-α-SynO. Data are expressed as means ± SD. *P* values are based on two-tailed unpaired *t* test. *N* = 7 animals per group (Vehicle and α-SynOs). *p ≤ 0.05.

Altered expression pattern of miRNAs have been implicated in various neurodegenerative processes, including retinal damage [36]. Therefore, after identification of miRNAs associated with PD, we constructed a miRNA-to-pathway network (Fig. 3A) to predict the miRNAs combinatorial effects on various targets and associated pathways known to be involved in PD. After inspecting the targets of each pathway and selecting those with at least one member of “TNF signaling pathway” as a miRNA target, we performed an inverse search to create a pathway-to-miRNA network (Fig. 3B). On the basis of the centrality metrics analysis of the two enriched networks (Fig. 3A, B), supported also by literature-based rescoring of the predicted list of putative miRNAs, we evaluated the expression of nine miRNAs (miR-128-2-5p, miR-128-3p, miR-384-5p, miR-155-5p, miR-146a-5p, miR-let7a-5p, miR-125b-5p, miR-27a-3p, miR-27a-5p) in the retinas of H-α-SynO-infused rats as compared to vehicle-treated rats. Interestingly, out of the nine miRNAs analyzed (Supplementary Table 1), real-time PCR analyses highlighted a statistically significant upregulation of three predicted PD-associated miRNAs, namely miR-27a-3p, miR-128-2-5p, miR-384-5p, in the retina of H-α-SynOs-infused rats (Fig. 3C).

### miR-384-5p-target molecule SIRT1 is reduced in the retina of H-α-SynOs-infused rats

**Fig. 4:**
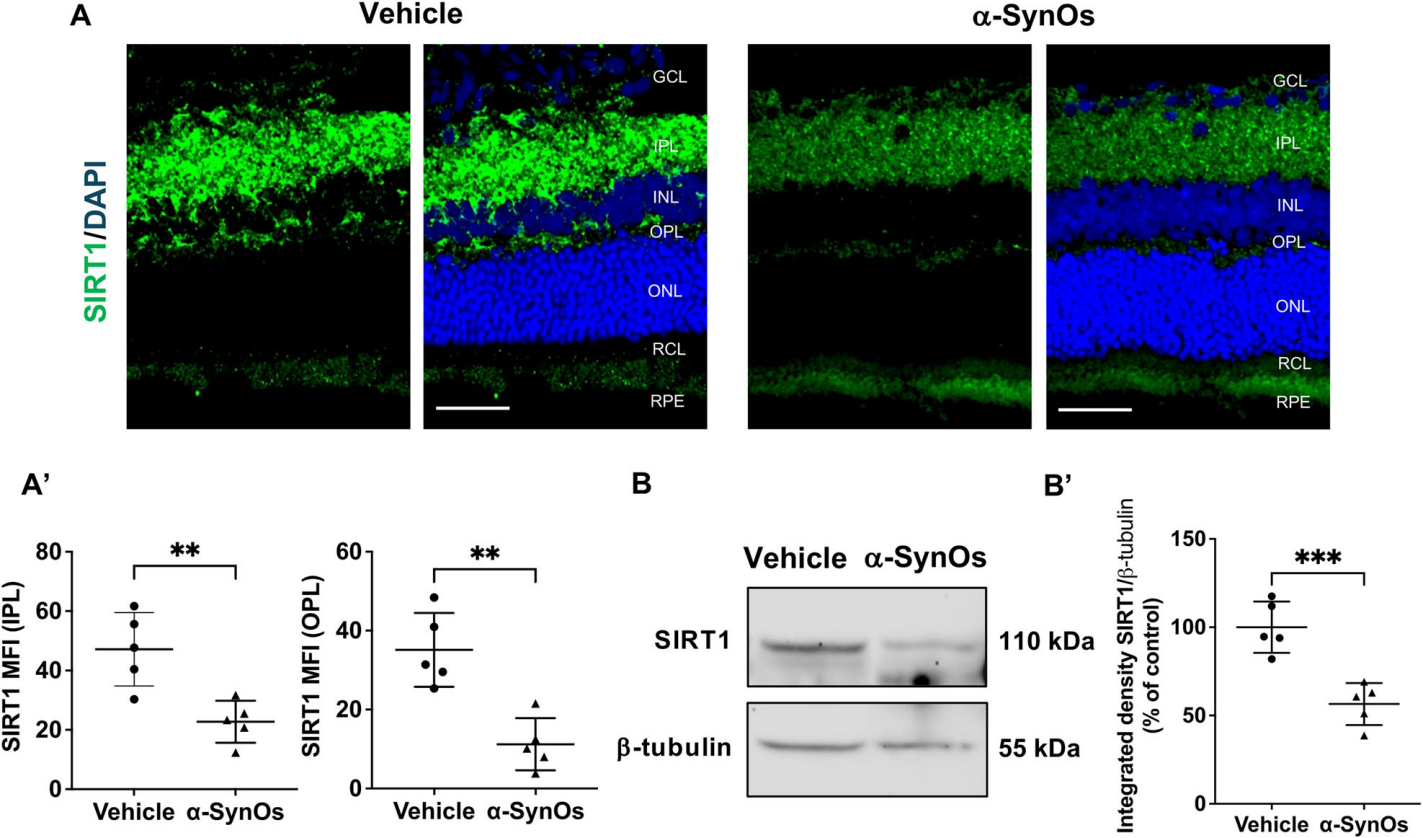
miR-384-5p-target molecule SIRT1 is reduced in the retina of H-α-SynOs-infused rats. **A** Confocal microscopy images of retinal slices stained with SIRT1 antibody. Original magnification, ×40. Scale bar = 50 µm. **A’** Quantification of SIRT1expression in the IPL and OPL from H-α-SynOs or Vehicle infused rats, presented as mean fluorescent intensity (MFI). Data are expressed as means ± SD. *P* values are based on two-tailed unpaired *t* test. *N* = 5 animals per group (Vehicle and α-SynOs); five independent retinal samples. **B** Representative western blot images of SIRT1 protein expression in retinal lysates from H-α-SynOs or Vehicle infused rats. **B’** Densitometric analysis of Western blots. Data are expressed as means ± SD. *P* values are based on two-tailed unpaired *t* test. *N* = 5 animals per group (Vehicle and α-SynOs); five independent retinal samples, two pooled retinas per sample of each group. **p ≤ 0.01, ***p ≤ 0.001.

Among the dysregulated miRNAs identified in our model, we focused on the upregulation of miR-384-5p expression in rats subjected to H-α-SynOs infusion. This microRNA has previously been found to play a crucial role in both in vitro and in vivo models of PD [37, 38]. Since Sirtuin 1 (SIRT1) is a validated (according to Tarbase v8 algorithm) [39] and a predicted target (according to microTG algorithm) [40] of miR-384-5p, we analyzed the retinal expression levels of SIRT1 protein. We observed a significant decrease in SIRT1 protein expression, compared to the vehicle-infused group (Fig. 4).

Immunofluorescence analysis revealed that such a reduction in SIRT1 was specifically detected in the IPL and OPL of the retinas in H-α-SynOs infused rats compared to vehicle-injected rats (Fig. 4A, A’). Furthermore, WB analysis confirmed the significant decrease in SIRT1 protein levels in the retina of H-α-SynOs infused rats relative to the controls (Fig. 4B, B’).

### Retinal macroglia and microglia activation in H-α-SynOs-infused rats

To better understand inflammatory changes in the retina, as a part of the broader spectrum of PD neuropathological processes regulated by SIRT1 and α-Syn accumulation [41, 42], we evaluated the extent of retinal macroglia and microglia activation by assessing the expression of glial fibrillary acidic protein (GFAP) and ionized calcium-binding adapter molecule 1 (Iba-1), respectively.

**Fig. 5:**
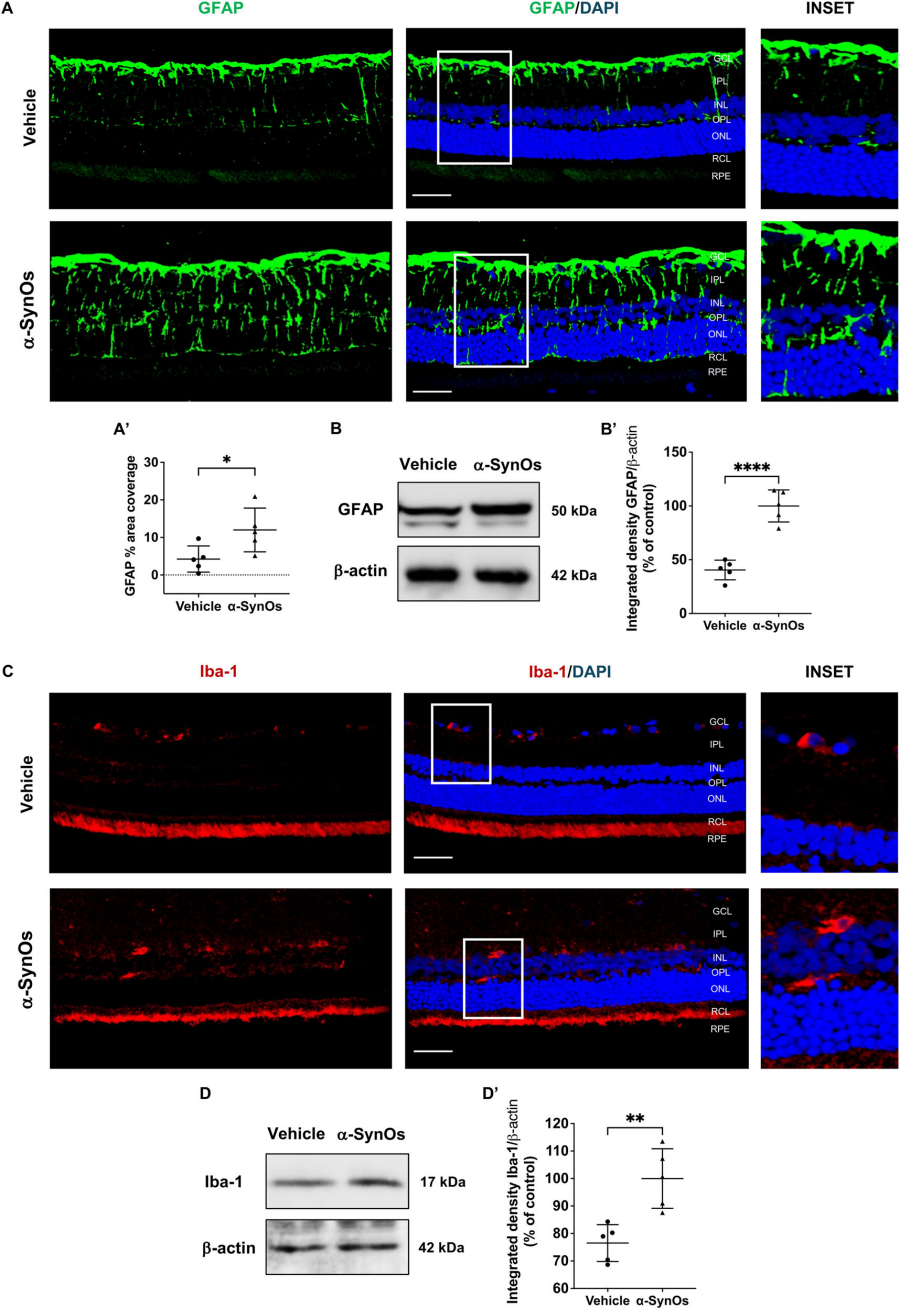
Enhanced retinal macroglia and microglia activation following intracerebral H-α-SynOs infusion. **A** Confocal microscopy images of GFAP-stained retinal slices. Original magnification, ×40. Scale bar = 50 µm. The inset represents the zoom-in area indicated in the full-scale image. **A’** Quantification of percent area coverage of GFAP staining in the retina from H-α-SynOs or Vehicle infused rats. Data are expressed as means ± SD. *P* values are based on two-tailed unpaired *t* test. **B** Representative western blot images of GFAP protein expression in retinal lysates from H-α-SynOs or Vehicle infused rats. **B’** Densitometric analysis of Western blots. Data are expressed as means ± SD. *P* values are based on two-tailed unpaired *t* test. **C** Confocal microscopy images of retinal slices stained with Iba-1 antibody. Original magnification, ×40. Scale bar = 50 µm. The inset represents the zoom-in area indicated in the full-scale image. **D** Representative western blot images of Iba-1 protein expression in retinal lysates from H-α-SynOs or Vehicle infused rats. **D’** Densitometric analysis of Western blots. Data are expressed as means ± SD. *P* values are based on two-tailed unpaired *t* test. For IHF *N* = 5 animals per group (Vehicle and α-SynOs); five independent retinal samples. For WB analysis *N* = 5 animals per group (Vehicle and α-SynOs); five independent retinal samples, two pooled retinas per sample of each group. *p ≤ 0.05, **p ≤ 0.001, ****p ≤ 0.0001.

Immunofluorescence analysis of retinal sections showed that GFAP immunoreactivity was virtually limited to the inner margin of the retina and mostly restricted to the ganglion cell layer (GCL) in vehicle-injected rats (Fig. 5A). In contrast, retinas of H-α-SynOs-infused rats exhibited intense GFAP immunoreactivity, with radiating brightly stained processes extending throughout the entire retinal thickness and to the outer nuclear layer (ONL) (Fig. 5A, A’).

GFAP protein expression was also assessed by WB analysis, which revealed a prominent expression in the retinas of H-α-SynOs-infused rats (Fig. 5B, B’).

Confocal microscopy images for Iba-1 immunostaining, while generally showing a low number of microglial cells in retinas of both Vehicle- and H-α-SynOs-infused rats, demonstrated a marked increase in Iba-1-positive cells particularly within the IPL and OPL of the retinas of H-α-SynOs-infused rats (Fig. 5C). On the other hand, Iba-1 immunoreactivity was less pronounced and primarily localized to the GCL in the retinas of Vehicle-treated rats (Fig. 5C), indicating a distinct, region-specific pattern of microglial activation in response to α-Syn infusion. Consistent with these observations, WB analysis revealed a significant increase in Iba-1 expression levels in the retina of H-α-SynOs-infused rats (Fig. 5D, D’).

### Activation of the TLR4/NF-κB axis and inflammatory response in the rat retina after intracerebral infusion of H-α-SynOs

**Fig. 6:**
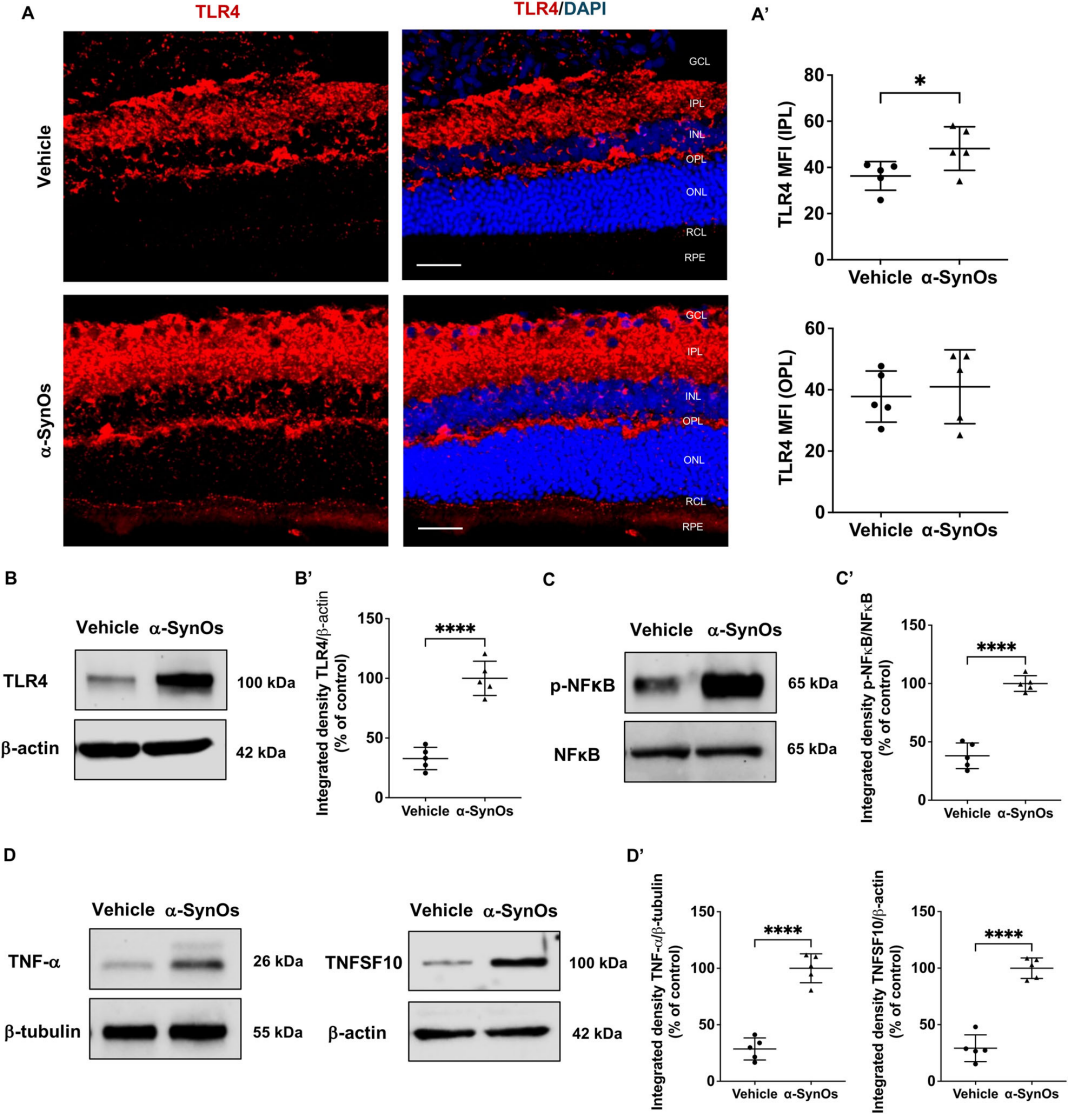
Activation of the TLR4/NF-κB axis and inflammatory response in the rat retinas following intracerebral H-α-SynOs infusion. **A** Confocal microscopy images of TLR4-stained retinal slices. Original magnification, ×40. Scale bar = 50 µm. **A’** Quantification of TLR4 expression in the IPL and OPL from H-α-SynOs or Vehicle infused rats, presented as mean fluorescent intensity (MFI). Data are expressed as means ± SD. *P* values are based on two-tailed unpaired *t* test. **B** Representative western blot images of TLR4 protein expression in retinal lysates from H-α-SynOs or Vehicle infused rats. **B’** Densitometric analysis of Western blots. **C** Representative western blot images of p-NFκB and NFκB proteins expression in retinal lysates from H-α-SynOs or Vehicle infused rats. **C’** Densitometric analysis of Western blots. **D** Representative western blot images of TNF-a and TNFSF10 proteins expression in retinal lysates from H-α-SynOs or Vehicle infused rats. **D’** Densitometric analysis of Western blots. Data are expressed as means ± SD. *P* values are based on two-tailed unpaired *t* test. For WB analysis *N* = 5 animals per group (Vehicle and α-SynOs); five independent retinal samples, two pooled retinas per sample of each group. *p ≤ 0.05, ****p ≤ 0.0001.

To further understand the inflammatory processes occurring in the retina of H-α-SynOs-infused rats, we investigated the activation of the TLR4/NF-κB axis. Confocal immunofluorescence analysis revealed a significant upregulation of TLR4 expression in the retina of H-α-SynOs-infused rats as compared to vehicle-infused rats. Such a heightened expression was particularly evident in specific retinal layers, particularly in the IPL of H-α-SynOs-infused rats (Fig. 6A, A’). Furthermore, WB results confirmed the increased TLR4 protein expression in retinas of H-α-SynOs-infused rats as compared to vehicle-infused rats (Fig. 6B, B’). The increased expression of TLR4 was also accompanied by downstream activation of the transcription factor NF-κB, as evidenced by the heightened expression of phosphorylated NF-κB (p-NF-κB) in the retina of H-α-SynOs-infused rats (Fig. 6C, C’).

Since TLR4/NF-κB pathway ultimately results in the induction of pro-inflammatory cytokines production [43], we next investigated the retina’s inflammatory cytokine profile. Specifically, WB analysis showed a significant increase in the expression of the inflammatory cytokines TNF-α and TNFSF10 (Fig. 6D, D’).

## Discussion

Several retinal alterations, including retinal thinning [44] and dopaminergic neuron dysfunction [14], have been observed in patients with PD. Notably, the retinas of PD patients frequently exhibit the accumulation of p-α-Syn [15, 16], a well-established marker of α-Syn aggregation, which mirrors the brain’s α-Syn pathology [1, 2, 45]. Such changes suggest that the retina, as an integral component of the CNS [10], may actively recapitulate the brain neuropathological processes associated with PD.

In the present study, we demonstrate that the infusion of toxic oligomeric α-Syn species into the rat SNpc induces multiple pathological alterations in the retina that largely resemble human PD retinal pathology, suggesting a causal role of toxic α-Syn in retinal impairment, and confirming the high toxicity of these protein aggregates [41]. Importantly, retinal changes were investigated in a well-characterized rat model of PD, previously shown to exhibit hallmark brain pathology, including dopaminergic neuron degeneration leading to motor deficits, deposition of p-α-Syn, and neuroinflammation in both motor and cortical regions [21–23, 42]. A pivotal advantage of the model used in this study lies in the high homogeneity of α-Syn species infused into the rat brain, in terms of structure and size, allowing for the assessment of the specific toxicity of oligomeric protein species [41]. Our results are in line with previous seminal studies reporting p-α-Syn aggregates in transgenic mice, such as the TgM83 and the Thy1-h[A30P]a-Syn mouse models [32, 46], which have shown a causal link between α-Syn overexpression and retinal pathology. However, these models lack a detailed prodromal profile, particularly with respect to key PD hallmarks, such as dopaminergic neuron dysfunction or inflammation in the retina and/or in the brain. This gap underscores the need for a model that effectively capture the retinal-brain correlation in PD. Moreover, other studies have reported retinal ganglion cells apoptosis and altered retinal thickness in a toxin-induced model of PD [47, 48].

Our findings show that the intranigral infusion of toxic H-α-SynOs disrupts the balance between α-Syn and p-α-Syn in the rat retina, with increased expression of p-α-Syn (pS129) particularly in synapse-rich layers such as the OPL and in the RPE, a layer essential for photoreceptor maintenance and visual function. Consistently, previous studies reported the preferential accumulation of phosphorylated α-Syn in the OPL, a synapse-rich region connecting photoreceptors, horizontal cells, and bipolar cells, where it causes severe degeneration and rod pathway dysfunction, contributing to visual deficits [49]. In such a scenario, our finding potentially suggests synaptic vulnerability, consistent with prodromal PD pathology. In parallel, p-α-Syn accumulation in the RPE [50], highlights how α-Syn toxicity compromises retinal homeostasis and contribute to the visual disturbances typical of PD. While a detailed investigation of transmission through the visual pathway falls outside the scope of this study, our findings, together with previous studies [21–23, 34], support the hypothesis that H-α-SynOs, once infused into the SNpc, may have triggered a spreading process, likely through the neural connectome. Consistent with the neuropathological features of proteinopathies such as PD, these toxic α-Syn species may recruit endogenous α-Syn, promoting its conversion into phosphorylated aggregates that progressively spread. This process could underlie the widespread pathology observed, reaching areas distant from the infusion site and even outside the brain, such as the retina [23].

Consistently with the pronounced expression of p-α-Syn in the retina of H-α-SynOs-infused rats, we also observed a significant reduction in the retinal expression of TH. While this result is suggestive of dopaminergic dysfunction, we acknowledge that a decrease in TH expression does not definitively imply neuronal death. Instead, it may reflect a loss of dopaminergic phenotype or functional suppression due to α-Syn-related toxicity. This observation extends previous evidence of dopaminergic alterations in the brain of the same PD model [21, 22], supporting the hypothesis that α-Syn toxicity is not confined to the nigrostriatal system but exerts widespread effects on CNS dopaminergic networks, including retinal projections.

At the molecular level, our study identifies the involvement of miRNAs, which are recognized as key regulators of PD pathogenesis [51–53]. Specifically, we observed a significant upregulation of three miRNAs predicted to be implicated in PD pathogenesis, namely miR-27a-3p, miR-128-2-5p and miR-384-5p in the retina of H-α-SynOs-infused rats. Notably, miR-27a-3p is predicted to target SRRM2, a protein that regulates spliceosome activity. Dysregulation of SRRM2 splicing has been reported in the PD brain [54], and both miR-27a-3p and SRRM2 have been described in peripheral leukocytes PD patients and proposed as early disease biomarkers [55]. In addition, an upregulation of miR-27a-3p has also been observed in the hippocampus of patients affected by Alzheimer’s disease [56], further supporting its involvement in neurodegenerative processes.

We also found a significant upregulation of miR-128-2-5p in the retina of our PD rat model, a miRNA known to regulate neuroinflammatory genes, and highly represented in peripheral immune cells of patients affected by neurodegenerative diseases [57].

Noteworthy, we found a significant upregulation of miR-384-5p and a concurrent reduction in expression level of its molecular target, SIRT1, in the retinas of rats infused with H-α-SynOs. This finding aligns with previous evidence indicating an uptick in the expression of miR-384-5p in dopaminergic neuronal-like SH-SY5Y cells treated with rotenone [37], a compound widely employed to model PD pathology. In our in vivo model of PD, we confirmed that miR-384-5p acts as a negative regulator of SIRT1 expression in the retina, reinforcing its role in PD-related retinal degeneration [58]. The observed downregulation of SIRT1 protein expression supports the hypothesis that miR-384-5p may contribute to retinal damage by suppressing neuroprotective pathways, given SIRT1’s established role in regulating inflammation and oxidative stress [59–62].

Building upon our understanding of SIRT1’s role in modulating the neuroinflammatory responses, we observed a pronounced macroglial and, to a lesser extent, microglial activation, in the retinas of H-α-SynOs-infused rats, as indicated by GFAP and Iba-1-immunoreactivity, respectively. Regarding the latter, while retinas from vehicle-treated rats showed limited Iba-1 immunoreactivity primarily localized in the GCL, retinas of H-α-SynOs-infused rats exhibited a marked increase in Iba-1-positive cells, particularly in the IPL and OPL, indicating a region-specific pattern. Such apparent discrepancy in microglial localization is likely due to the dynamic nature of retinal microglia, which can become activated and redistribute across different retinal layers in response to pathological stimuli [63]. Importantly, these changes occurred in the absence of a direct retinal exposure to α-SynOs, indicating that the inflammatory response was a secondary effect of the CNS infusion, and that brain-derived α-Syn toxicity may propagate the inflammatory signal to distant neural structures such as the retina. These findings align with previous studies linking H-α-SynOs toxicity to neuroinflammatory responses in several brain regions, including those related to motor and cognitive functions [21, 22, 42].

Such a sustained inflammatory environment, alongside the presence of p-a-Syn [64], was coupled with a significant increase of TLR4 expression and downstream activation of the NF-κB transcription factor in the retinas of H-SynO-infused rats. Dysregulation of TLR4, a member of TLR family expressed on cells of the innate immune system including glial cells, plays a crucial role in alpha-synucleinopathies [65] by amplifying glial pro-inflammatory responses triggered by α-Syn aggregates [65, 66]. Thus, the heightened expression of astrocytic and microglial markers, coupled with TLR4/NF-κB axis activation, highlights the pivotal role of innate immune responses in driving retinal inflammation. Indeed, a sustained activation of the TLR4/NF-κB pathway contribute to amplify the retinal pro-inflammatory profile in PD, as evidenced by a marked increase in the expression of downstream pro-inflammatory cytokines, such as TNF-α and TNFSF10, observed in the retina of H-α-SynOs infused rats.

Collectively, our study provides compelling evidence that intracerebral infusion of H-α-SynOs initiates a cascade of multiple retinal alterations that recapitulates key pathological features observed in PD patients. Specifically, intracerebral infusion of H-α-SynOs induces the accumulation of p-α-Syn in several retinal layers, reduces TH expression in dopaminergic cells potentially reflecting phenotypic impairment, alters the expression of PD-related miRNAs (notably miR-384-5p), and suppresses the neuroprotective factor SIRT1. These molecular shifts promote a pro-inflammatory state, as evidenced by the upregulation of TLR4 and NF-κB signaling and the increased expression of cytokines like TNF-α and TNFSF10. We hypothesize that these components interact in a feed-forward loop, wherein α-Syn aggregation, miRNA imbalance, and immune activation reinforce one another, driving retinal dysfunction. Although mechanistic dissection was not the primary focus of this study, our data highlight several pathways potentially involved in α-Syn-induced retinal dysfunction. While further work is required to establish direct causality and assess therapeutic potential, these initial insights provide a valuable framework for future investigation.

Altogether, these findings not only emphasize the retina as a valuable site for monitoring brain neurodegenerative processes in PD, but also offer new insights into the molecular and cellular mechanisms driving retinal pathology. Our study bridges a crucial gap between preclinical models and clinical observations, reinforcing the retina’s potential as a surrogate site for monitoring disease progression and exploring mechanistic targets in neurodegeneration.

## Materials and methods

### Animals and stereotaxic surgery

34 male Sprague–Dawley rats (275–300 g, ENVIGO RMS S.R.L., San Pietro al Natisone, Italy) were housed in standard conditions of temperature (21 ± 1 °C) and humidity (60%) under a 12 h light/dark cycle (lights on 7:00 A.M) with access to standard chow and water ad libitum.

After deep anesthesia with Fentanyl (3 mg/kg) and medetomidine hydrochloride (0.35 mg/kg), rats at 3 months of age were stereotaxically injected with 5 µL of H-α-SynOs into the SNpc (coordinates relative to bregma; −5.4 mm anteroposterior; ±1.9 mm from the midline; −7.2 mm beneath the dura) bilaterally at the rate of 1 µL/min via a silica microinjector (*n* = 17). Control animals, indicated as vehicle, received sterile phosphate buffer saline (PBS) (*n* = 17). Animals were assigned randomly to the different experimental groups.

Three-months after H-α-SynOs infusion one group of animals was deeply anesthetized and transcardially perfused in ice-cold 0.1 M PBS (pH 7.4) followed by 4% formaldehyde (*n* = 5 from each experimental group) for immunofluorescence analysis. Fresh retinal tissues were collected from a separate group of animals (*n* = 12 from each experimental group), which were also transcardially perfused with ice-cold PBS prior to tissue collection and stored at −80 °C for subsequent molecular analysis.

All procedures were performed in accordance with the ARRIVE guidelines and with the guidelines and protocols approved by the European Community (2010/63UE L 276 20/10/2010). Experimental protocols were approved by the Italian Ministry of Health (authorization N. 766/2020-PR). Every endeavor was undertaken to minimize animal suffering and discomfort while also reducing the number of experimental animals employed.

### Production of recombinant H-α-Syn

Recombinant human α-synuclein (H-α-Syn) was purified in *E. coli* using plasmid pT7-7 encoding for the protein as previously described [41]. Briefly, protein expression was induced with 1 mM IPTG at 37 °C for 4 h. The cell lysate was centrifuged at 22,000 × *g* (Beckman Coulter, Brea, CA, USA) for 30 min, and the supernatant was then heated for 20 min at 70 °C. After an additional centrifugation at 22,000 × *g*, 10 mg·mL^−1^ streptomycin sulfate was added to the supernatant for DNA precipitation performing two steps of precipitation and centrifugation. Subsequently, 360 mg·mL^−1^ ammonium sulfate was added to the supernatant to precipitate the recombinant H-αSyn. The obtained pellet was resuspended in 25 mM Tris–HCl, pH 7.7 and, after dialysis against the same buffer, loaded onto an anion exchange column (26/10 Q sepharose high performance, GE Healthcare, Little Chalfont, UK) to be eluted with a 0–1 M NaCl step gradient. Further purification was achieved by applying size exclusion chromatography (Hiload 26/60 Superdex 75 preparation grade, GE Healthcare). The purity of the sample was analyzed by SDS-PAGE, and the protein concentration was determined from the absorbance at 275 nm using an extinction coefficient of 5600 M^−1^·cm^−1^.

### Purification of H-α-SynOs

Toxic oligomeric samples were prepared from purified recombinant H-α-Syn as previously described [41]. Lyophilized protein was resuspended in PBS buffer at a pH of 7.4 and a concentration of 12 mg·mL^−1^, then passed through a 0.22 μm cutoff filter before incubation at 37 °C for 24 h without agitation. Residual fibrillar species were removed by ultracentrifugation for 1 h at 288,000 × *g*, and excess of monomers were removed using several filtration steps with 100 kDa cutoff membranes. At the end of the purification procedure, and prior to intracerebral inoculation, oligomers were tested for endotoxin contamination via the LAL (Limulus Amebocyte Lysate) assay (Kairosafe, Italy).

### Bioinformatics analysis

To identify microRNAs (miRNAs) putatively dysregulated in the retina of our rat PD model, we conducted a literature search to identify miRNAs associated with PD. Then, we identified the rat homologous sequence (rno-miR-128-2-5p; rno-miR-128-3p; rno-miR-384-5p; rno-miR-155-5p; rno-miR-146a-5p; rno-miR-let7a-5p; rno-miR-125b-5p; rno-miR-27a-3p; rno-miR-27a-5p). Subsequently, using DIANA-miRPath v.3 (accessed on May 2023), we analyzed these miRNAs dysregulated in PD to predict their combinatorial effects on different targets, and then associated pathways known to be implicated in the disease.

A miRNA-to-pathway network based on DIANA-miRPath v.3 was constructed and analyzed using Cytoscape v.3.7.0. Thereafter, targets of each pathway identified in the preliminary query of DIANA-miRPath were inspected and filtered for those involving at least one member of the TNF signaling pathway. Subsequently, an inverse search with DIANA-miRPath v.3 was performed to build a pathway-to-miRNA network. Centrality metrics analysis of networks was carried out using Cytoscape v.3.7.0.

### microRNA extraction, cDNA synthesis, and qPCR

Retinas were extracted from the ocular globes and placed in RNAlater solution (Ambion Biosystems, Austin, TX, USA). Samples were stored at 4 °C overnight and subsequently transferred to −80 °C. Total RNA extraction from rat retina samples was conducted using TRIzol Reagent (Invitrogen, Life Technologies, Carlsbad, CA), following the manufacturer’s guidelines.

To increase RNA yields, 5 μg of glycogen was added to the aqueous phase of each sample. The A260/A280 ratio of the optical density of RNA samples was measured with Nanodrop 1000 spectrophotometer (ThermoFisher Scientific Inc., MA, USA). For cDNA synthesis, 20 ng of total RNA was retrotranscribed with TaqMan® microRNA Reverse Transcription Kit (ThermoFisher Scientific Inc., Cat. No. 4366596), as per the manufacturer’s instructions. MiRNA expression was evaluated by Taqman® MicroRNA Assays (ThermoFisher Scientific Inc., Cat. No 4427975) and Taqman® Universal Mastermix II no UNG (ThermoFisher Scientific Inc., Cat. No 4440043). The following microRNAs (miRNAs) were analyzed: rno-miR-128-2-5p (assay ID: 464165_mat), rno-miR-128-3p (assay ID: 002216), rno-miR-384-5p (assay ID: 002602), rno-miR-155-5p (assay ID: 002571), rno-miR-146a-5p (assay ID: 000468), rno-let-7a-5p (assay ID: 000377), rno-miR-125b-5p (assay ID: 000449), rno-miR-27a-3p (assay ID: 000408), rno-miR-27a-5p (assay ID: 002445). The miR-16-5p (assay ID: 000391) was used as an endogenous control for the normalization. Real-time PCR was carried out on a 7,900 HT Fast Real Time PCR System (Applied Biosystems, Monza, Italy). Differentially expressed miRNAs were identified by calculating expression fold changes by the 2 − ΔΔCt method.

### Tissue homogenization and protein extraction

Retinas isolated from rats injected with H-α-SynOs and control rats were dissected in ice-cold Hank’s balanced salt solution (HBSS: 137 mM NaCl, 5.4 mM KCl, 0.45 mM KH2PO4, 0.34 mM Na2HPO4, 4 mM, NaHCO3, 5 mM glucose; pH 7.4). For protein extraction, retinal tissues were lysed in RIPA buffer containing protease and phosphatase inhibitor mixture (ThermoFisher Scientific Inc.) and sonicated on ice (three pulses of 2 s each). After sonication, the homogenates were centrifuged at 14,000 rpm for 10 min at 4 °C and the supernatant was collected. The protein concentration of the supernatant was determined using the Bradford method [67].

### Western blot analysis

Equal amounts of protein extracts (40 µg) were resolved on 8–12% SDS-PAGE gels and transferred onto Hybond ECL nitrocellulose membranes (GE Healthcare, Little Chalfont, UK). The membranes were blocked for 1 h at RT with 5% nonfat dry milk or 5% BSA in phosphate-buffered saline plus 0.1% Tween 20 (PBS-T). For primary antibody reactions, a rabbit anti-α-Syn (Cell Signaling Technology Inc., Danvers, MA, USA), or a rabbit anti-p-α-Syn (Abcam, Cambridge, UK), or a mouse anti-TH (Merck Millipore, Burlington, MA, USA), or a rabbit anti-SIRT1 (Santa Cruz Biotechnology Inc., Santa Cruz, CA, USA), or a mouse GFAP (Cell Signaling Technology Inc.), or a rabbit Iba-1 (ThermoFisher Scientific Inc.), or a mouse TLR4 (Santa Cruz Biotechnology Inc.), or a rabbit anti-NFκB p65 (Santa Cruz Biotechnology Inc.), or a rabbit anti-p-NFκB p65 (Santa Cruz Biotechnology Inc.), or a goat anti-TNF-α antibody (Santa Cruz Biotechnology Inc.), or a rabbit anti-TNFSF10, were added to membranes and incubated overnight at 4 °C on an orbital shaker. Subsequently, the membranes were washed with PBS-T and probed for 1 h at RT with the appropriate horseradish peroxidase-conjugated anti-rabbit or anti-mouse or anti-goat IgG antibody (Amersham Life Science, Buckinghamshire, UK). Beta-tubulin or β-actin (Santa Cruz Biotechnology Inc.) were used as control to verify the amount of protein loaded in the gels. Following another round of washing with PBS-T, the protein bands were visualized using enhanced chemiluminescence (ThermoFisher Scientific Inc.) and captured using the iBright FL1500 Imaging System (ThermoFisher Scientific Inc.). Densitometric analysis of band intensity on immunoblots was performed using IMAGE J software (https://imagej.nih.gov/ij/). See *Original Data* for full and uncropped WBs. Detailed information about the antibodies used are reported in Supplementary Table 2.

### Immunofluorescence staining

After collection, eye globes were fixed in 4% w/v paraformaldehyde solution in PBS with a pH of 7.4 for 2 h at room temperature. Paraffin-embedded retinal tissues were sliced into 5 μm sections and placed on glass slides. After deparaffinization and rehydration, tissue specimens were treated as previously described [68] with some adjustments. Briefly, after antigen retrieval in sodium citrate buffer (10 mM sodium citrate, 0.05% Tween-20, pH 6.0) through microwave treatment for 15 min, slides were washed twice for 5 min in PBS containing PBS containing 0.025% Tween-20 (PBS-T). Subsequently, they were blocked in 3% BSA in PBS-T for 1 h at RT in an humid chamber, and then incubated overnight at 4 °C with BSA 1% with the following primary antibodies: a rabbit anti-α-Syn (Cell Signaling Technology Inc.), or a rabbit anti-p-α-Syn (Abcam), or a mouse anti-TH (Merck Millipore), or a rabbit anti-SIRT1 (Santa Cruz Biotechnology Inc.), or a mouse GFAP (Cell Signaling Technology Inc.), or a rabbit Iba-1 (ThermoFisher Scientific Inc.), or a mouse TLR4 (Santa Cruz Biotechnology Inc.).

For fluorescence detection of the antigens of interest, after three washes in PBS of 5 min each, sections were incubated using the appropriate fluorescent-labeled secondary antibodies (ThermoFisher Scientific Inc.) for 1 h at RT in the dark (See Supplementary Table 2 for the detailed list of antibodies used). Finally, for staining of nuclei and stabilization of fluorescent signals, slides were washed with PBS, covered with mounting medium with DAPI (F6057, Fluoroshield; Sigma-Aldrich, Milan, Italy) and secured with a coverslip. Digital images were acquired using a confocal laser scanning microscope (TCS SP8, Leica Microsystems GmbH, Wetzlar, Germany), using 40× objective and were processed using ImageJ software. Mean Fluorescence Intensity (MFI) was used to evaluate the intensity of the immunofluorescence signal. Briefly, after converting the images to 16-bit, inverting to grayscale, and using the appropriate threshold function, the mean gray value of the images was measured [69]. To evaluate the percent area coverage of GFAP, images were first converted to 16-bit grayscale, and using the appropriate threshold function, the positive stained area in the images was measured. For TH^+^ cell counting, after converting single-color images to 16-bit grayscale and applying the appropriate threshold to highlight the structures of interest, binary images were created. Positively stained cells were counted using the “Analyze Particles” function.

### Brain TH immunohistochemistry and stereology

After perfusion, brains were post-fixed overnight in 4% paraformaldehyde-PBS and stored in 0.1% NaN3-PBS at 4 °C. Subsequently, 40-µm-thick serial sections of the midbrain were cut using a vibratome. Sections were blocked with normal donkey serum and then incubated with a polyclonal rabbit anti-TH (1:1000, Millipore, Burlington, MA, USA) primary antibody. The reaction was amplified using a biotinylated secondary antibody and visualized using the avidin-biotin-peroxidase complex (ABC, Vector, UK) method, with 3,3’-diaminobenzidine (Sigma-Aldrich, St. Louis, MO, USA) as a chromogen.

TH-immunoreactive neurons were counted bilaterally in the SNPc, as previously described [70]. The counting was performed using a specialized software (Stereologer, System Planning and Analysis, Inc., Alexandria, VA, USA), which was linked to a motorized stage on a BX-60 Olympus light microscope (Olympus, Segrate, Italy). The total number of TH-positive cells was estimated using the optical fractionator method. This method combines the optical dissector with the fractionator sampling scheme, providing an unbiased estimation of the number of 3-D objects, regardless of their shape, size, and orientation [71]. Systematic random sampling of cells within the area of interest was achieved using the “Stereologer” software, which generated equidistant counting frames (frame area = 50 μm²). The sampling fraction was defined at low magnification, and cells were sampled using a ×40 oil immersion objective through a specified depth with a 2 μm guard zone. The coefficient of error (CE) for each estimation and animal ranged from 0.05 to 0.1.

### Statistical analysis

Investigators that carried out treatment and analyses were blinded to group labels; group identities were revealed only after graph design and completion of statistical analyses. A low difference between the means of groups and homogeneous variance within the groups was expected and sample size was determined based on power analysis calculations using G*power software [72]. Criteria for exclusion of animals or corresponding samples included weight loss exceeding 20% and signs of severe distress. However, within the monitoring of animal health during the experiment, no animals or samples met the exclusion criteria during the study. Graph design and statistical analyses were carried out with Graphpad prism version 9.0 (https://www.graphpad.com/scientific-software/prism/). After testing normality and homogeneity of variance of the data, a parametric (homoscedastic or Welch corrected) or non-parametric unpaired *t*-test was applied. Data were represented as means ± Standard deviation (SD), from at least three independent samples, and three technical replicates.

Supplementary information is available at Cell Death Discovery website.

## Supplementary information

Original Data

Supplementary Table 1

Supplementary Table 2

Supplementary Fig. 1

Supplementary figure legends

## Data Availability

The dataset used and/or analyzed in the current study are available from the corresponding author on request.
