# Supplementary material for: Retinal alterations resemble brain pathology in a rat model of Parkinson’s disease induced by intranigral infusion of α-synuclein oligomers
Source: Cell Death Discov. 2025 Nov 28;11:550. doi: 10.1038/s41420-025-02830-0 (PMC12663455; doi:10.1038/s41420-025-02830-0)
Supplement: Supplementary file 2 — Supplementary Table 1 [file 41420_2025_2830_MOESM2_ESM.docx]

**Supplementary Table 1. Full list of miRNAs analyzed in the retina of the PD rat model**

| **miRNAs** | **FC** | **t test** |
| --- | --- | --- |
| let-7a-5p | 1,069318 | 0,5112 |
| miR-27a-3p | 1,385057 | **0,0204** |
| miR-27a-5p | -1,15967 | 0,3107 |
| miR-125b-5p | -1,01051 | 0,8743 |
| miR-128-2-5p | 1,223332 | **0,0286** |
| miR-128-3p | -1,04626 | 0,8132 |
| miR-146a-5p | 0,898651 | 0,8798 |
| miR-155-5p | 0,811538 | 0,7623 |
| miR-384-5p | 1,108397 | **0,0314** |
