# Supplementary material for: Retinal alterations resemble brain pathology in a rat model of Parkinson’s disease induced by intranigral infusion of α-synuclein oligomers
Source: Cell Death Discov. 2025 Nov 28;11:550. doi: 10.1038/s41420-025-02830-0 (PMC12663455; doi:10.1038/s41420-025-02830-0)
Supplement: Supplementary file 3 — Supplementary Table 2 [file 41420_2025_2830_MOESM3_ESM.docx]

**Supplementary Table 2. List of antibodies and relative dilution used for western blot (WB) analysis and immunofluorescence (IHF)**

| Antibodies | Diluition for WB | Diluition  for IHF | Company (Catalog#) |
| --- | --- | --- | --- |
| Mouse monoclonal anti-TH | 1:500 | 1:200 | Merck Millipore (MAB318) |
| Mouse monoclonal anti-β-actin | 1:1000 | - | Santa Cruz Biotechnology (sc-47778) |
| Mouse monoclonal anti-TLR4 | 1:500 | 1:200 | Santa Cruz Biotechnology (sc-293072) |
| Goat polyclonal anti-TNFα | 1:200 | - | Santa Cruz Biotechnology (sc-1351) |
| Rabbit polyclonal Anti-TNFSF10 | 1:200 | - | Abcam (ab2435) |
| Mouse monoclonal anti-GFAP | 1:1000 | 1:200 | Cell Signaling Biotechnology (#3670) |
| Mouse monoclonal anti-β-tubulin | 1:1000 | - | Santa Cruz Biotechnology (sc-5274) |
| Rabbit polyclonal anti-α-Synuclein | 1:1000 | 1:200 | Cell Signaling Biotechnology (#2628) |
| Rabbit polyclonal anti-p-α-Synuclein (S129) | 1:1000 | 1:200 | Abcam (ab51253) |
| Rabbit polyclonal anti-NFκB p65 | 1:500 | - | Santa Cruz Biotechnology (sc-109) |
| Rabbit polyclonal anti-SIRT1 | 1:500 | 1:200 | Santa Cruz Biotechnology (sc-15404) |
| Rabbit polyclonal anti-p-NFκB p65 (Ser536) | 1:1000 | - | Cell Signaling Biotechnology (#3033) |
| Rabbit polyclonal anti-Iba-1 | 1:500 | 1:200 | ThermoFisher Scientific (PA527436) |
| Alexa Fluor 488 goat anti-mouse IgG | - | 1:500 | ThermoFisher Scientific (#A28175) |
| Alexa Fluor 546 donkey anti-mouse IgG | - | 1:500 | ThermoFisher Scientific (#A10036) |
| Alexa Fluor 488 goat anti-rabbit IgG | - | 1:500 | ThermoFisher Scientific (#A11008) |
| Alexa Fluor 546 donkey anti-rabbit IgG | - | 1:500 | ThermoFisher Scientific (#A10040) |
|  |  |  |  |
