# Supplementary material for: Retinal alterations resemble brain pathology in a rat model of Parkinson’s disease induced by intranigral infusion of α-synuclein oligomers
Source: Cell Death Discov. 2025 Nov 28;11:550. doi: 10.1038/s41420-025-02830-0 (PMC12663455; doi:10.1038/s41420-025-02830-0)
Supplement: Supplementary file 5 — Supplementary figure legends [file 41420_2025_2830_MOESM5_ESM.docx]

**Supplementary Figure Legends**

**Supplementary figure 1:** Nigral degeneration after H-α-SynOs infusion. A) Representative images of TH immunostaining in the SNpc of Vehicle and H-α-SynO-infused rats. (Magnification 5X; scale bar: 500µm). B) Stereological quantification of TH-stained cells in the SNpc. Data are expressed as means ± S.E.M. P values are based on two-tailed unpaired t test. ****p ≤ 0.0001.
